# Resting-state fMRI in Obsessive Compulsive Disorder patients compared to healthy controls

**DOI:** 10.1192/j.eurpsy.2025.523

**Published:** 2025-08-26

**Authors:** S. Chahal, P. Sharan, S. Kumaran, G. Hans

**Affiliations:** 1 PSYCHIATRY, GREATER MANCHESTER MENTAL HEALTH FOUNDATION TRUST, MANCHESTER, United Kingdom; 2 PSYCHIATRY; 3 Nuclear Magnetic Resonance, AIIMS, New Delhi, New Delhi, India

## Abstract

**Introduction:**

Previous resting-state fMRI studies have found hypoconnectivity between the areas underlying default mode and salience networks in OCD patients. A general dysconnectivity has been observed between the frontoparietal network and corticostriatal-thalamocortical loops in patients with OCD, We conducted a study to understand the neural correlates of OCD and its sub-types and compared them with healthy controls. As a part of the analysis of fMRI data, we also analyzed the resting state data for OCD patients and compared it to that of healthy controls.

**Objectives:**

To study the neural correlates of OCD using functional MRI by comparing the resting state functional connectivity in OCD patients with healthy controls.

**Methods:**

We used the resting state functional MRI data of 8 OCD patients and compared it with 10 healthy controls. The healthy controls and patients were not age—and sex-matched. The resting state fMRI data was assessed using the CONN functional connectivity toolbox, version 15.d, in MATLAB. The Regions of interest (ROIs) were mapped using the MNI coordinate system. The functional connectivity (FC) was studied with ROI-to-ROI analysis and seed-to-voxel analysis.

**Results:**

1. In ROI to ROI analysis between resting state networks, only one significant result was found when FC between all the brain networks was compared. as shown in table 1 and image 1.

**Table 1.** 

Seed	Target	T-statistic	X, Y, Z	p-uncorrected	p-FDR corrected
Lateral Occipital Cortex, inferior division Right (iLOCr)	Salience rostral pre-frontal cortex	T(16) = 5.32	46, -74, -2	0.0001	0.0112

2. Seed-to-voxel-based analysis revealed that at p-FWE <0.05 corrected, the left and right occipital pole and right intra-calcarine cortex were more active in OCD patients as shown in image 2.

**Image 1:**

**Figure:**
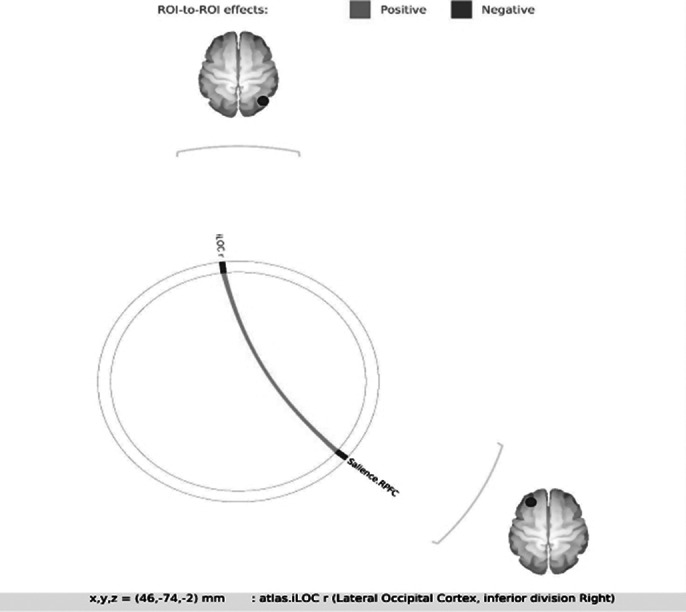

**Image 2:**

**Figure:**
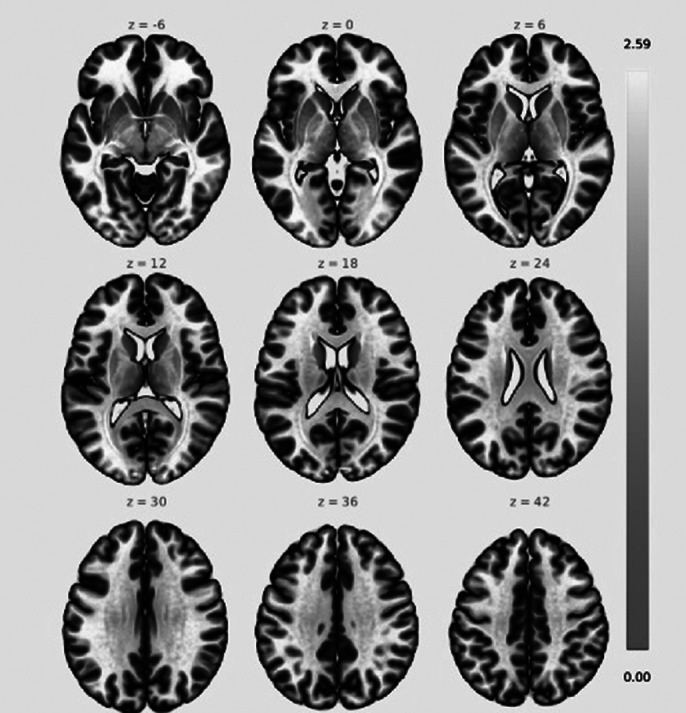

**Conclusions:**

1. ROI TO ROI analysis: Attempts to gain control over automatic processes are taxing on the cognitive resources and effective control is lacking in OCD, which could explain higher functional connectivity in the areas linked to having selective attention for specific visual stimuli as found in our study. The lateral occipital cortex supports both visual perception and multisensory integration and visual cortices have been seen to contribute to impulsivity and disorders commonly associated with impulsivity. The rostral prefrontal area in the salience network is involved in sequence selection and evaluation and has a possible role in attention. DMN and corticostriatal networks were normal possibly due to the small sample size, patients on treatment, etc.

2. Seed to Voxel Analysis showed left as well as right occipital poles and right intra-calcarine cortex which process visual stimuli, were more active at rest in OCD patients. Some studies have highlighted deficits in visuospatial processing in OCD patients. The intra-calcarine cortex would require further research for a better understanding of its role in OCD patients.

**Disclosure of Interest:**

None Declared

